# Nutrigenetic Screening Strains of the Mulberry Silkworm, *Bombyx mori*, for Nutritional Efficiency

**DOI:** 10.1673/031.012.0301

**Published:** 2012-01-22

**Authors:** Ramesha Chinnaswamy, Hothur Lakshmi, Savarapu S. Kumari, Chebba M. Anuradha, Chitta S. Kumar

**Affiliations:** ^1^ Silkworm Breeding and Molecular Genetics Laboratory, Andhra Pradesh State Sericulture Research and Development Institute, Kirikera-515 211, Hindupur, AP, India; ^2^ Silkworm Genetics and Breeding Laboratory, Central Sericultural Research and Training Institute, Central Silk Board, Berhmpore, 742 101, West Bengal, India; ^3^ Biology Division, Indian Institute of Chemical Technology, Tarnaka, Hyderabad - 500 007, AP, India; ^4^ Department of Biotechnology, College of Engineering and Technology, Sri Krishnadevaraya University, Anantapur- 515 003, AP, India; ^5^ Bioinformatics Centre, Department of Biochemistry, Sri Krishnadevaraya University Anantapur-515 003, AP, India

**Keywords:** approximate digestibility, breed, consumption index, leaf-cocoon ratio, leaf-shell ratio, metabolic rate, silkworm

## Abstract

The activity of sericulture is declining due the reduction of mulberry production area in sericulture practicing countries lead to adverse effects on silkworm rearing and cocoon production. Screening for nutrigenetic traits in silkworm, *Bombyx mori L*. (Lepidoptera: Bombycidae) is an essential prerequisite for better understanding and development of nutritionally efficient breeds/hybrids, which show less food consumption with higher efficiency conversion. The aim of this study was to identify nutritionally efficient polyvoltine silkworm strains using the germplasm breeds RMW_2_, RMW_3_, RMW_4_, RMG_3_, RMG_1_, RMG_4_, RMG_5_, RMG_6_ and APM_1_ as the control. The 1^st^ day of 5^th^ stage silkworm larvae of polyvoltine strains were subjected to standard gravimetric analysis until spinning for three consecutive generations covering three different seasons on 19 nutrigenetic traits. Highly significant (*p* ≤ 0.001) differences were found among all nutrigenetic traits of polyvoltine silkworm strains in the experimental groups. The nutritionally efficient polvoltine silkworm strains were resulted by utilizing nutrition consumption index and efficiency of conversion of ingesta/cocoon traits as the index. Higher nutritional efficiency conversions were found in the polyvoltine silkworm strains on efficiency of conversion of ingesta to cocoon and shell than control. Comparatively smaller consumption index, respiration, metabolic rate with superior relative growth rate, and quantum of food ingesta and digesta requisite per gram of cocoon and shell were shown; the lowest amount was in new polyvoltine strains compared to the control. Furthermore, based on the overall nutrigenetic traits utilized as index or ‘biomarkers’, three polyvoltine silkworm strains (RMG_4_, RMW_2_, and RMW_3_) were identified as having the potential for nutrition efficiency conversion. The data from the present study advances our knowledge for the development of nutritionally efficient silkworm breeds/hybrids and their effective commercial utilization in the sericulture industry.

## Introduction

The mulberry silkworm, *Bombyx mori* L. (Lepidoptera: Bombycidae) is a monophagous insect that feeds exclusively on the mulberry (*Morus spp.*) foliage for its nutrition and produces the natural proteinous silk. Intensive and cautious domestication over centuries has apparently privileged this commercial insect the opportunity to increase in nutrition efficiency. Nutritional intake has direct impact on the overall genetic traits such as larval and cocoon weight, amount of silk production, pupation, and reproductive traits. The sericulture activity is declining due the reduction of mulberry production area in sericulture practicing countries on silkworm rearing and silk production. This consensus is more pronounced in countries more advanced in sericulture compared to developing countries in Asia and Pacific regions. Thus, among many factors attributed to reduction in silk production, the major one is the lack of nutrition efficiency conversion in polyvoltine silkworm strains in tropical areas compared to bivoltine strains. Therefore, one of the key considerations in developing polyvoltine hybrids for tropical regions could be the need for nutrition efficiency conversion in polyvoltine strains. The recent advances in silkworm breeding and those with nutrition efficiency conversion have opened up new avenues to evolve nutritionally efficient productive silkworm hybrids (Hamano et al. 1986; Mano et al. 1991; Ding et al. 1992; Tzenov et al. 1999; Zhang et al. 2002; Rahmathulla et al. 2004; Ramesha et al. 2010).

Sericulture in India is practiced predominantly in tropical environmental regions such as Karnataka, Andhra Pradesh, Tamil Nadu, West Bengal, and to a limited extent in the temperate environment of Jammu and Kashmir. The existing situation provides scope for creating polyvoltine × bivoltine hybrids (crossbreeds) as a commercial venture as hybrids above 90% of total silk production (Ramesha et al. 2009a).

Crossbreed nutritional efficiency conversion is low when compared to the existing bivoltine (Ramesha et al. 2010). During the last few decades, a number of silkworm hybrids have been developed (Chandrashekharaiah and Ramesh Babu 2003) and selected for exploitation at the field level. These productive breeds are not as nutritionally efficient as bivoltine silkworm breeds and originated from indigenous strains. Because India is a major tropical country in the production of crossbreeds under limited area of mulberry production, estimation of phenotypic stability for high nutritional efficiency conversion is considered one of the most important aspects for sustainable progress in polyvoltine breeding.

Earlier studies have demonstrated fundamental interaction of nutrition/physiology on gene expression (Giacobino et al. 2003; Phillips et al. 2008). Similarly, nutrition or diet/physiology play imperative roles in insect gene expression (Yocum et al. 2006; Rharrabe et al. 2010), but have no implications in selection of parental resources for breeding programs. Some earlier studies addressed the importance of nutritional aspects, but nutrigenetics is often neglected in the selection of silkworm breeds in respect to nutrition consumption and efficiency conversion for identifying the nutritionally efficient silkworm breeds/hybrids. However, a clear understanding of the genetic basis and variability in the gene expression of quantitative traits during the analysis of nutrigenetic traits are an important step for the selection of potential nutritionally efficient polyvoltine parental resources for breeding programs.

The purpose of this study is to obtain new data about screening for the nutritional efficiency in polyvoltine silkworms, not only to augment current knowledge on gene interaction between nutrition efficiency conversion and quantitative traits under varied conditions, but also to provide valuable information that will allow identification of nutritionally efficient polyvoltine silkworm breeds based on the standard gravimetric analysis relative to 19 important economical nutrigenetic traits as ‘biomarkers’.

## Materials and Methods

### Polyvoltine silkworm breeds

The 8 polyvoltine silkworm germplasm breeds used were RMW_2_, RMW_3_, RMW_4_, RMG_3_, RMG_1_, RMG_4_, RMG_5_, and RMG_6_. These strains, with varied phenotypic quantitative traits and non-hibernating nature, maintained at gene bank of Silkworm Breeding and Molecular Genetics Laboratory, Andhra Pradesh State Sericulture Research and Development Institute, Hindupur, India, were utilized for the study along with the popular silkworm breed APM_1_ as the control.

### Silkworm rearing

The disease-free eggs from each strain were reared and cocoons were harvested and maintained until eclosion of moths. Healthy female moths emerging on the peak day of eclosion were allowed to mate for 3–4 hours and held until oviposition. The eggs were incubated at 25 ± 1 ^°^C temperature and 70– 80% RH after surface treatment with 2% formalin solution. 20 to 30 eggs were chosen from each brood and pasted onto to egg sheets. Three such egg sheets for each breed were prepared, wrapped in white tissue paper and boxed with black paper to synchronize the embryonic development. On the day of hatching, the eggs were exposed to light in order to obtain uniform hatching, and freshly chopped mulberry leaves were fed to the young larvae. The whole process from silkworm egg incubation to completion of rearing activities was carried out under hygienic conditions in a silkworm-rearing house that had been thoroughly disinfected with bleach followed by formalin solution. Silkworm rearing was conducted for each breed in plastic trays by feeding them the V_1_ variety of mulberry leaves from the wellmaintained irrigated mulberry garden on campus. A standard rearing procedure was adopted as recommended by Krishnaswami et al. (1973). The young larvae (1^st^–3^rd^ instars) were reared at 26–28 °C with 80–90% RH, and late age larvae (4^th^ and 5^th^ instars) were maintained at 24–26 °C with 70–80% RH until the resumption of 4^th^ molt. Each batch was divided into two, one of which was maintained as reserved stock under standard rearing conditions, and the second was subjected to standard gravimetric analysis.

### Estimation of nutritional traits

The nutrigenetic traits estimation study was carried out between February 2007 and January 2008 covering pre-monsoon, monsoon, and post-monsoon of the year in a completely randomized block design. Silkworm rearing was conducted following the standard method under the recommended temperature and relative humidity until the 4^th^ molt. On the 1^st^ day of fifth instar, 50 healthy silkworm larvae per breed in three replications of 150 larvae each were selected for estimation on nutritional traits analysis. Accurately weighed fresh mulberry leaves were fed 3 times a day to the experimental batch and the control. Simultaneously, an additional batch of larvae for each breed was maintained to determine the dry weight on subsequent daily increments in larval weight were recorded separately as suggested by Maynard and Loosli (1962). Silkworm rearing continued using appropriate plastic trays. The healthy larvae were counted daily in each replicate, and any missed larvae were replaced from the reserve batch. Left over leaves and excreta were collected on each subsequent day , separated manually and dried in a hot air oven daily at about 100 ^°^C until they reached constant weight using an air-tight electronic balance . When the larvae finished feeding they were shifted to the mountage for spinning at normal ambient temperature of 25 ± 2 ^°^C and RH 65 ± 5%. Cocoons were harvested 4–5 days later after completion of cocoon spinning. Harvested cocoons were accessed for quantitative traits using the equations detailed below. The dry weight of left over leaves, excreta, larvae, cocoon, and shell in each of the breed was recorded. The nutrigenetic traits interaction was obtained by utilizing standard gravimetric analysis methods for three consecutive seasons.

During the silkworm nutritional study, data were collected on the biomass of larvae and cocoons for the 19 nutrigenetic traits on ingesta, digesta, excreta, approximate digestibility (AD), reference ratio (RR), consumption indices (CI), relative growth rate (RGR), respiration and metabolic rate (MR), efficiency conversion of ingesta (ECI) and digesta (ECD) for larva, cocoon, and shell. Further, the ingesta and digesta required for producing one gram of cocoon and shell (I/g and D/g) were collected and calculated as described by standard gravimetric methods (Waldbauer 1968; Scriber and Feeny 1979; Kogan and Parra 1981; Slansky and Scriber 1985), the equations with brief description of the nutrigenetic traits evaluated given below.

### Ingesta (g). 

Total intake of the dry weight (g) of mulberry leaves by silkworm larvae during the 5^th^ stage up to spinning or ripening stage: (Dry weight of leaf fed — Dry weight of left over leaf).

### Digesta (g). 

Total assimilated dry food from the intake or ingesta of dry weight of mulberry leaves by silkworm larva during the 5^th^ stage until spinning or ripening: (Dry weight of leaf ingested — dry weight of litter).

### Excreta (g). 

Refers to the non-utilized mulberry leaves in the form of litter from the ingested mulberry leaves of a silkworm: (Ingesta — Digesta).

### Approximate digestibility 

(%). Directly indicates the assimilation efficiency of mulberry leaves and depends on the passage rate of food through gut in silkworm: (AD = Dry weight of Digesta / Dry weight of food ingested × 100).

### Reference ratio. 

An indirect expression of absorption and assimilation of food. Expresses the ingesta required per unit excreta produced: (RR= Dry weight of food ingested / Dry weight of excreta).

### Consumption index. 

Relates the rate of food intake to the mean weight of the larvae during the feeding period: (CI = Ingesta / 5^th^ stage mean fresh larval weight (g) × 5^th^ stage larval duration in days)

### Relative growth rate. 

Refers to larval gain biomass and indicates the efficiency of conversion of nutrition into larval biomass: (RGR = Weight gain of the larva during feeding period / 5^th^ stage mean fresh larval weight (g) × 5^th^ stage larval duration in days)

### Respiration. 

A catabolic reaction in which total oxidation of the digested or assimilated food for releasing energy required for the entire biological activities by break down of macromolecules into simpler molecules: (Dry weight of food digested — Maximum dry weight of larvae).

### Metabolic rate. 

Measure of total biochemical reactions involving both catabolic and anabolic reactions of an organism, associated with the degradation of macromolecules into smaller unit and vice versa: (MR= Respiration / 5^th^ stage mean fresh larval weight (g) × 5^th^ stage larval duration in days).

### Efficiency conversion of ingesta to larva

(%). Associated with the efficiency conversion of ingested nutrition into biomass or body matter at different stages and expressed in percentage. ECI to larva was the efficiency of conversion of ingested food into larva: (ECI larvae = Maximum dry weight of larva / Dry weight of ingesta × 100).

### Efficiency conversion of digesta to larva

(%). The expression of efficiency conversion of digesta into larval biomass: (ECD larvae = Maximum dry weight of larva / Dry weight of digesta × 100).

### Efficiency conversion of ingesta to cocoon

(%). This is the most economically important trait used by the sericulture industry. It was the expression of efficiency conversion of ingesta into cocoon, also referred to as the leaf-cocoon conversion rate. This nutrigenetic trait was kept as the ultimate index for assessing the superiority of breed for nutritional efficiency in this investigation: (ECI cocoon = Dry weight of cocoon / Dry weight of ingesta × 100).

### Efficiency conversion of digesta to cocoon

(%). It was the expression for efficiency conversion of digesta into cocoon: (ECD cocoon = Dry weight of cocoon / Dry weight of digesta × 100).

### Efficiency conversion of ingesta to shell 

(%). This was the expression efficiency conversion of ingesta into shell. It is also referred to as the leaf-shell conversion rate and is the ultimate index to evaluate superiority of breed in nutritional efficiency: (ECI shell = Dry weight of shell / Dry weight of ingesta × 100).

### Efficiency conversion of digesta to shell

(%). The expression of efficiency conversion of digesta into shell: (ECD shell = Dry weight of shell / Dry weight of digesta × 100).

### Ingesta per gram cocoon (g). 

This was another important trait of economical significance to assess silkworm breed performance in nutrigenetic analysis. It was the expression of total ingesta required for the production of one gram of cocoon: (I/g cocoon = Dry weight of ingesta / Dry weight of cocoon).

### Digesta per gram cocoon (g). 

The total digesta requisite for the production of one gram of cocoon: (D/g cocoon = Dry weight of digesta / Dry weight of cocoon).

### Ingesta per gram shell (g). 

The total ingesta requisite for the production of one gram of shell: (I/g shell = Dry weight of ingesta / Dry weight of shell).

### Digesta per gram shell (g). 

The total digesta requisite for the production of one gram of shell: (D/g shell = Dry weight of digesta / Dry weight of shell).

The data on nutritional traits of both the experimental and control breed were recorded on 19 nutrigenetic traits for each replicate. Data was subjected to ANOVA statistical analysis with assistance of the computer packages developed by Indostat Service Pvt. Ltd.

## Results

### Morphological features of polyvoltine germplasm breeds

The morphological differences were found between germplasm breeds with respect to origin, egg, larva, and cocoon traits. All nine breeds originated from Madagascar. The chorion of 5 breeds was pigmented and 3 were mixed, and serosa was white in 3 breeds (RMW_2_, RMW_3_, RMW_4_) and yellow in 6 breeds (RMG_1_, RMG_3_, RMG_4_, RMG_5_, RMG_6_, APM_1_). All of these polyvoltine breeds were non-hibernating in nature. The larval marking of all breeds was plain. The larval body build of 6 breeds was slender, whereas 2 were stout. Cocoon shape in 8 breeds spun oval except for RMW_4_, whose cocoons was elongated and oval in shape. Cocoon grains were fine in six breeds and coarse in 2 breeds. The popular commercial polyvoltine silkworm breed APM_1_ was originally from Andhra Pradesh State Sericulture Research and Development Institute, Kirikera-505 211, Hindupur, Andhra Pradesh, India with non-hibernating nature, whose egg chorian color was brown with yellow egg shell. APM_1_ had plain larval marking, slender body shape, and an oval shaped cocoon with greenish yellow color of medium grains.

### Performance on nutrigenetic traits

Considerable variation was found for 19 nutrigenetic traits among the polyvoltine breeds on nutritional parameters. Data were obtained for ingesta, digesta, excreta, AD, RR, CI, RGR, respiration, MR, ECI, and ECD to larval biomass, ECI and ECD to cocoon and shell, I/g and D/g to cocoon and shell for nine polyvolitne breeds under standard nutritional estimation including control breed. There was evidence of clear declines in consumption of mulberry leaf, but efficiency in food conversion to biomass for major nutrigenetic traits in all experimental polyvoltine breeds over the control breed (Tables 1 and 2).

### Ingesta, digesta, excreta, approximate digestibility, and reference ratio

Among the experimental breeds, the lowest ingesta was in RMG_4_ followed by RMW_2_, RMW_3_, and RMG_6_, compared with control APM_1_ .Digesta for the control APM_1_ was intermediate compared to RMG_1_ and RMG_4_. The highest excreta was found in RMG_6_, and the lowest was found in RMG_1_, while the control APM_1_ measured 1.928 g. Approximate digestibility (AD) ranged from 38.61% to 32.75%. Reference ratio value was the highest in RMG_1_, followed by the control APM_1_ and RMG_4_.

### Consumption index, relative growth rate,respiration, and metabolic rate

The highest consumption index was found in control APM_1_, followed by RMW_2_ and RMG_4_. Maximum relative growth rate was shared in RMG_1_, RMG_4_, and RMG_5_, and the minimum in RMG_3_ was observed among polyvoltine germplasm breeds. Relatively high respiration was found in RMG_1_ followed by the control APM_1_ and RMG_4_. Metabolic rate was highest in RMG_1_ and lowest in RMG_4_ (Table 1).

### Efficiency of conversion of ingesta and digesta to larval biomass

The efficiency of mulberry leaf ingested and digested in conversion to silkworm larval biomass or body varied prominently among the polyvoltine breeds (Table 2). The highest efficiency conversion of ingesta for larva was recorded in RMG_4_ followed by RMW_2_, RMG_6_, and APM_1_. Efficiency conversion of digesta to larva varied significantly, and more efficient conversion of digested food into larval biomass was recorded in RMG_4_ followed by RMW_2_ and RMG_6_.

### Efficiency of conversion of ingesta and digesta to cocoon and shell

The highest efficiency of conversion to cocoon was shown in RMG_4_, followed by RMW_2_, APM_1_, and RMG_6_. The efficiency conversion of digesta to cocoon was highest in RMG_4_ and lowest in RMG_1_. The efficiency conversion of ingesta to shell ranged from 7.29% in RMG_4_ to 6.01% in RMG_6_. Similarly, efficiency conversion of digesta to shell was highest in RMG_4_, followed by RMW_2_ and RMG_1_ (Table 2).

### Ingesta and digesta per gram to cocoon and **shell**

The highest ingesta required to produce 1 gram of cocoon was found in RMG_6_, while the lowest was found in RMG_4_. Similarly, the amount of digesta required to produce 1 gram of cocoon was highest for RMG_1_ and lowest for RMG_4_. Ingesta required to produce 1 gram of shell was higher for RMG_6_ and the control, than for RMG_4_. The highest digesta required for 1 gram of shell was found in RMG_1_, and the lowest was found in RMW_2_ (Table 2).

### Percentage difference on consumption traits over control

The percentage of difference among experimental breeds and control was highest in RMG_6_ and lowest in RMG_4_ for the trait of ingesta/larva. RMG_1_ and RMG_4_ showed highest and lowest percent of difference for the traits of digesta/larva. A significant difference resulted between RMG_6_ and RMG_1_ for excreta/larva. With respect to approximate digestibility, a positive difference in RMG_1_ and negative difference in RMG_4_ was found. A large difference between RMG_1_ and RMG_4_ was found for the reference ratio. High positive differences in RMG_5_ and low in RMG_3_ were found for the relative growth ratio. With respect to respiration, there was a positive difference in RMG_1_ and a negative difference in RMG_4_. The highest difference for metabolic rate was found in RMG_1_, and the lowest was found in RMG_4_ (Table 3).

### Percentage difference on efficiency conversion traits over control

Percentage difference in efficiency conversion traits among experimental breeds over control was highest in RMG_4_ and lowest in RMG_6_ for the ECI/larva trait. RMG_4_ and RMG_4_ showed high and low percent of difference for the traits of ECD/larva, respectively. A significant difference resulted between RMG_4_ and RMG_6_ for ECI/cocoon. With respect to ECD/cocoon, a positive difference in RMG_4_ and a negative difference in RMG_1_ was found. A positive difference was found for ECI/shell in RMG_4_, and a negative difference was found in RMG_6_. A high positive difference was found in RMG_4_, while a negative difference was found in RMG_3_ on ECD/shell (Table 4).

### Percentage difference on nutrition requisite per gram cocoon and shell over control

With respect to I/g cocoon, a significant difference between RMG_6_ (2.16%) and RMG_4_ was found. A positive difference in RMG_1_ and a negative difference in RMG_4_ was found for the trait of D/g cocoon. A positive difference was found in RMG_6_ and a negative difference was found in RMG_4_ for I/g shell. A high positive difference was found in RMG_1_, whereas there was a negative difference in RMG_4_ for D/g shell (Table 4).

### CI and nutritional efficiency traits as a ‘biomarker’

**Figure 1.**
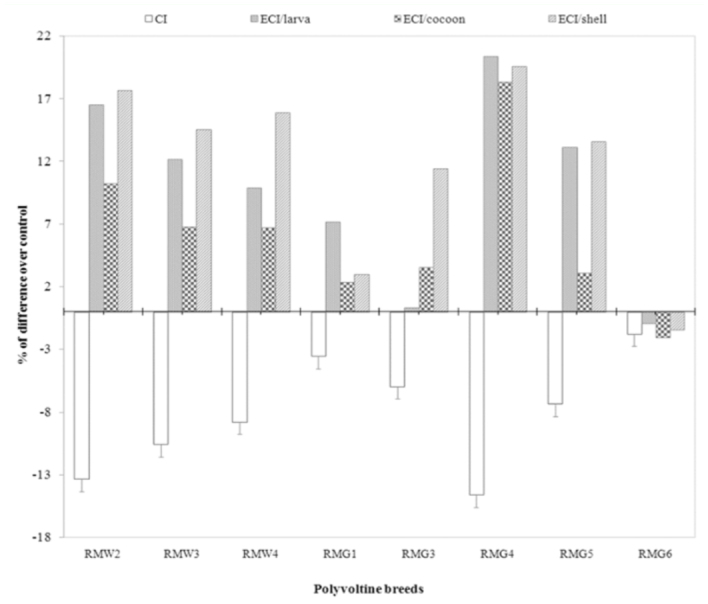
Comparison of consumption and efficiency conversion traits on biomass in polyvoltine breeds over control. High quality figures are available online.

The nutritional efficiency conversion contributes directly to the major portion of the cost benefit ratio of silkworm rearing, and is considered an important physiological criterion for evaluating the nutritional superiority of silkworm breeds. The efficiency of conversion of ingesta (ECI) to cocoon and shell, which are otherwise referred as leafcocoon and leaf-shell ratios, are the ultimate indices to evaluate nutritional efficiency of a silkworm breed or hybrid in terms of the production of cocoon/shell. Comparing nutrition consumption to efficiency of conversion, all of the breeds were grouped for relative nutritionally efficiency. Among them, three experimental polyvoltine breeds had less CI with higher efficiency conversion over control, and were considered to be nutritionally efficient (Figure 1). The minimum consumption index and maximum efficiency of conversion/cocoon were both observed in RMG_4_ with respect to the control. This was followed by RMW_2_ and RMW_3_ with respect to control. Four polyvoltine breeds were found to exhibit < 10% CI, ranging from -3.53% (RMG_1_) to -8.79% (RMW_4_), and ECI/cocoon ranging from 2.36% to 6.71%, and were considered to be moderately nutritionally efficient. RMG_6_ was identified as less nutritionally efficient or nutritionally inefficient, as it showed < 2% of CI with ECI/cocoon of-2.08% with respect to control (Tables 3 and 4). By considering CI and ECI/cocoon as the indices or ‘biomarkers’ for nutritional efficiency, the polyvoltine breeds were classified as nutritionally inefficient (RMG_6_ ), moderately nutritionally efficient (RMW_3_, RMW_4_, RMG_3_, RMG_5_ ), and nutritionally efficient (RMG_4_, RMW_2_, RMW_3_) for nutrition consumption and efficiency conversion traits. With respect to percentage difference between the experimental polyvoltine breeds over control breed, negative correlations were found against 10 traits and positive correlations were found in nine traits (Figures 4 and 5).

### Seasonal variation on nutrigenetic traits

**Figure 2.**
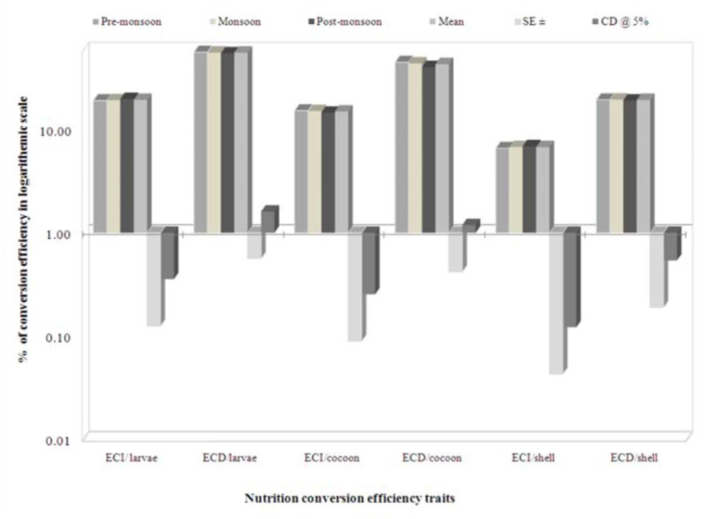
Seasonal variance on nutritional efficiency conversion traits in logarithmic scale on biomass in polyvoltine silkworm breeds. High quality figures are available online.

Considering seasonal variance, performance during the post-monsoon season resulted in higher values for most of the nutritional consumption traits except for the reference ratio, which was followed by monsoon. Additionally, lower values were exhibited during pre-monsoon season and standard error (SE) was zero for relative growth rate (Table 5). A maximum of 19.29% efficiency of conversion of ingesta to larval biomass during post-monsoon, and a minimum of 19.02% in pre-monsoon was found. The highest efficiency of conversion of digesta to larval biomass in pre-monsoon (55.96%) was followed by monsoon (55.27%) and postmonsoon (54.19%). The highest efficiency of conversion of ingesta and digesta to cocoon were observed during pre-monsoon, followed by monsoon and post-monsoon. Highest efficiency of conversion of ingesta to shell was noticed during post-monsoon (6.88%), followed by monsoon (6.74%) and premonsoon (6.58%). The highest efficiency of conversion of digesta to shell was found during monsoon (19.43%), followed by premonsoon (19.36%) and post-monsoon (19.01%). The higher standard errors and critical difference at 5% were noticed for the traits of efficiency of conversion of digesta/larva and efficiency of conversion of digesta/cocoon compared to efficiency of conversion of digesta/shell on a logarithmic scale among nutrition conversation efficiency traits (Figure 2).

**Figure 3.**
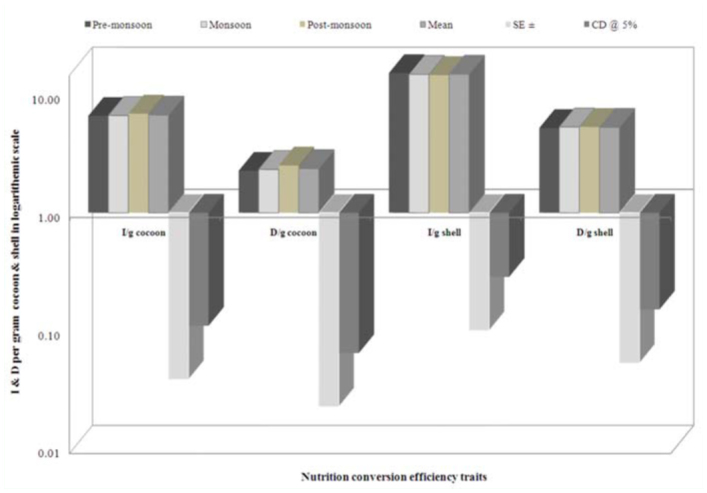
Seasonal variance on nutrition requisite per gram cocoon and shell in logarithmic scale in polyvoltine breed. High quality figures are available online.

The highest ingesta/g cocoon requisite was found during post-monsoon (6.92%), followed by monsoon (6.67%) and pre-monsoon (6.62%). Similarly, more digesta/g cocoon was found during post-monsoon (2.51%), followed by monsoon (2.33%) and premonsoon (2.27%). A significant conversion efficiency was found for ingesta/g shell during pre-monsoon (15.26%), followed by monsoon (14.89%) and post-monsoon (14.68%). More digesta/g shell was shared during monsoon and post-monsoon seasons (5.34%) than premonsoon (5.22%). The higher standard errors and CD at 5% were noticed in ingesta/g shell on a logarithmic scale among nutrition requisite per gram of cocoon and shell (Figure 3).

**Figure 4.**
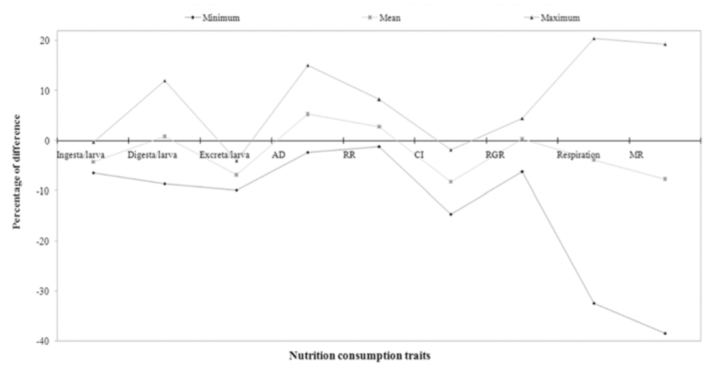
Percentage difference among the nutrition consumption traits on polyvoltine breeds over control. High quality figures are available online.

The collected data for the 19 nutrigenetic traits were statistically analyzed by the variance test for significant differences between the experimental breeds over control in different seasons. Results obtained after analysis of variance of mean squares revealed highly significant (*p* ≤ 0.001) differences among all nutrigenetic traits for the breeds. Further, amongst seasons, highly significant (*p* ≤ 0.001) differences were observed among major nutrigenetic traits except in four efficiency conversion traits. Highly significant differences in standard error (SE) and critical difference (CD) at 5% was demonstrated for the trait of approximate digestibility and lower in relative growth rate among consumption traits (Figures 6). Similarly, a significant difference in SE and CD at 5% was shown for the trait of ECD to larva, followed by ECD to cocoon and was lowest in D/g cocoon among nutritional conversion efficiency traits (Figure 7). All major economically important nutrigenetic traits showed a decline in consumption of mulberry leaves with more conversion efficiency into biomass compared to the control (Figure 1).

**Figure 5.**
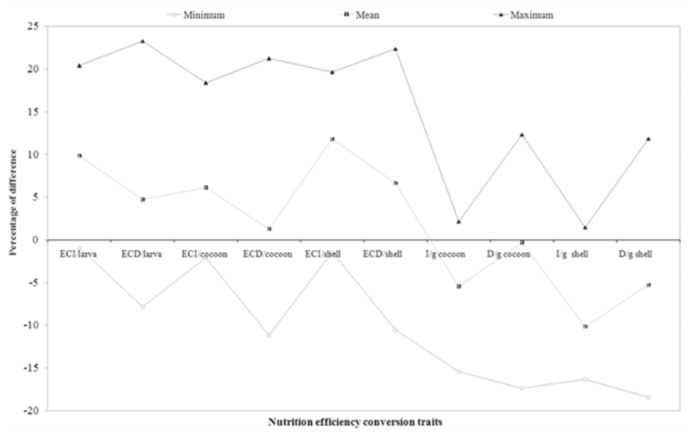
Percentage difference among the nutrition efficiency conversion traits on biomass in polyvoltine breeds over control. High quality figures are available online.

**Figure 6.**
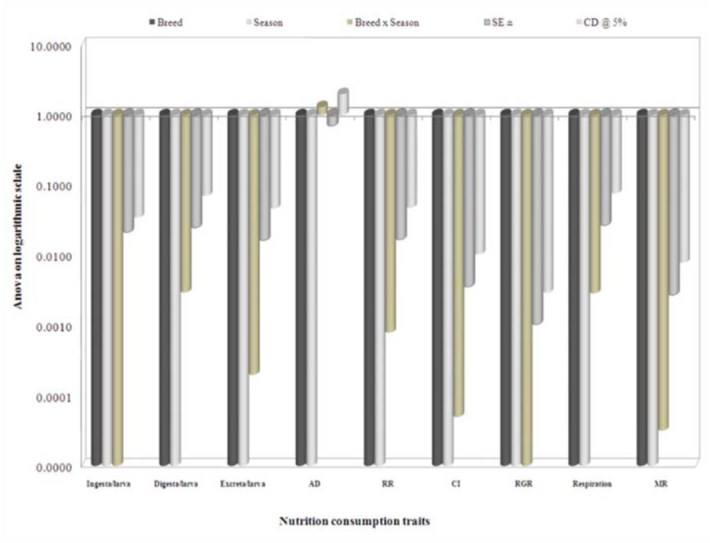
Analysis of variance in logarithmic scale on nutrition consumption traits with seasons among polyvoltine breeds. High quality figures are available online.

### Discussion

Based on all morphological and nutrigenetic traits, and lower consumption of mulberry leaves and maximum efficiency of conversion of nutrients, with highly significant (p ≤0.001) differences among polyvoltine genotypes and seasons for 19 nutrigenetic traits, three polyvoltine silkworm breeds, RMG_4_, RMW_2_, RMW_3_, were identified as potential nutritionally efficient breeding resources for breeding programs.

**Figure 7.**
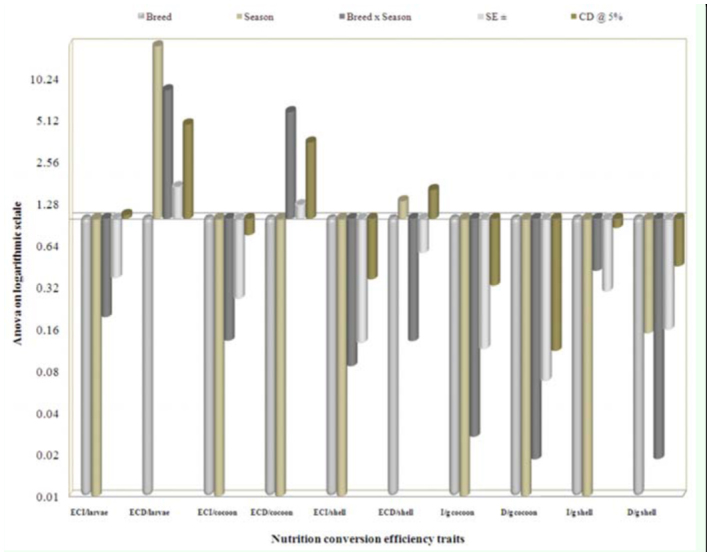
Analysis of variance on logarithmic scale on nutrition efficiency conversion traits with seasons among polyvoltine breeds. High quality figures are available online.

Silkworm breeding can be defined as the science of improving the genetic entity of silkworms in relation to their economic utility. Silk producing countries in Asia and Pacific regions experience serious problems in the field of silkworm breeding. This investigation intends to serve as a guideline to organize or revive breeding programs, as well as a quick reference to silkworm breeders. It also offers a brief background on silkworm breeding, including genetics, nutrition, and physiology. It also outlines the necessary facilities and tools required to establish modern silkworm breeding programs for the sustenance of sericulture in the tropical regions. A great diversity of the mulberry silkworm *B*. *mori* L. exists globally from which several silkworm breeds have evolved by selection and cross breeding. Quantitative genetics helps in the study of the inheritance of polygenic traits among related individuals. In silkworm populations subject to artificial selection, genetic parameters are required to be estimated to formulate breeding plans (Talebi et al. 2010; Xu et al. 2011).

The study of the interactions between nutrition and quantitative traits, the major genetic traits of silkworm showed a greater decline in consumption with increases of food efficiency conversion into biomass in experimental polyvoltine breeds compared to the control. A similar result was reported for polyvoltine and commercial hybrid silkworms by Maribashetty et al. (1999) and Meneguim et al. (2010) respectively. Such dietary factors and related metabolic interactions on specific gene expression were also reported by Walker and Blackburn (2004). Nutrition affects nearly all biological processes including the rates of biochemical and physiological reactions (Parra and Kogan 1981; Paul et al. 1992), and eventually can affect the larval quality or quantity of cocoon crops in the silkworm. Several reports (Ueda 1965; Mano et al. 1991; Paul et al. 1992; Rahmathulla et al. 2005) demonstrated that silkworms were more responsive to nutrition supplement during the 4^th^ and 5^th^ stages, which are recommended for the recognition and selection of nutritional efficient silkworm breeds for commercial purposes. Hence, the nutrition utilization study was confined to the 5^th^ instar larvae, since 80–85% of total leaf consumption was observed in this stage of silkworm development (Ueda 1965; Rahmathulla et al. 2005). For instance, polyvoltine breeds reared in tropical countries are known to be less nutritionally efficient, which is also true with cross breeds that have evolved for a tropical climate (Rahmathulla et al. 2003; Ramesha et al. 2009b; Ramesha et al. 2010). It is essential to analyze nutrigenetic traits to understand the racial difference among polyvoltine germplasm breeds before hybrid preparation for commercial purposes. Recently, the effects of nutrigenetic traits for bivoltine germplasm breeds also have been shown by Ramesha et al. (2010).

The success of the sericulture industry depends upon several factors, including production of quality mulberry leaves. This factor is of vital importance, since it accounts for 60% of total expenditures (Datta and Nanavaty 2005). It is well understood that the majority of the economically important genetic traits of silkworm are qualitative in nature, and phenotypic expression is greatly influenced by the environmental factors such as temperature, relative humidity, light, and nutrition (Krishnaswami et al. 1971; Wu and Hau 1993; Tazima 1984; Thaigarajan et al. 1993; Zhang et al. 2002; Zhao et al. 2007; Ramesha et al. 2010). Therefore, it is essential to gauge the degree of phenotypic difference of the economical traits to understand the genetic steadiness under the controlled nutrition conditions and the productivity of polyvoltine breeds. The problem of balancing and fixing the desirable traits for a given environment is a challenging task for the silkworm breeder. Hence, understanding the range of a reaction of the selected breeds to variable nutritional conditions is important for the breeder to utilize them appropriately in hybrid programs. Breeders in the field agree that it is a difficult task to breed such polyvoltine breeds that are more nutritionally efficient with productive traits. It is a wellestablished fact that tropical polyvoltine strains are inefficient in nutrition conversion even with proper management during rearing. In order to better select the breeds with high efficiency conversion of nutrition, it is important to analyze the impact of nutrigenetic traits on many silk-yielding attributes, the genetic traits of silkworm breeds, and their heritability.

Maximum values were observed for CI, respiration, and metabolic rate among polyvoltine germplasm breeds during premonsoon season. It was noticed that the low ingesta with high consumption index and relative growth rate might be due to shorter larval duration in pre-monsoon than the other two seasons (Gokulamma and Reddy 2005). Such fluctuation is indicative of the seasonal effect on nutrigenetic traits.

The nutritional efficiency of conversion contributes directly to the major portion of the cost benefit ratio of silkworm rearing, and is considered an important physiological criterion for evaluating the nutritional superiority of silkworm breeds. The efficiency of conversion of ingesta to cocoon and shell, which are otherwise referred to as leaf-cocoon and leaf-shell ratios, are the ultimate indices to evaluate nutritional efficiency of a silkworm breed or hybrid in terms of the production of the cocoon/shell (Ding et al. 1992; Junliang and Xiaofeng 1992; Maribashetty et al. 1999; Prabhakar et al. 2000; Ramesha et al. 2010).

In order to achieve greater success in this regard, it is important to understand the level of nutrition efficiency in polyvoltine silkworm breeds. The main objective of this study was to identify polyvoltine silkworm breeds with nutrition efficiency among eight polyvoltine breeds evaluated for 19 important nutrigenetic traits. The results obtained for conversion index and efficiency of conversion of ingesta to biomass through standard gravimetric method for three successive generations on different seasons is supported by earlier observations (Hassanein et al. 1972; Sumioka et al. 1982; Anantharaman et al. 1994; Trivedy and Nair 1999; Kumaresan et al. 2000). Our emphasis was on the phenotypic manifestation of 19 nutrigenetic traits. The results revealed highly significant (*p* ≤ 0.001) variability among the polyvoltine breeds with respect to 19 nutrigenetic traits over control for the experimental germplasm breeds. Although earlier studies showed that some polyvoltine breeds are moderately nutritionally efficient (Remadevi et al. 1992; Datta et al. 1996; Maribashetty et al. 1999), our study indicated that seven polyvoltine breeds were identified as nutritionally efficient by lower consumption index (-14.58% to -3.53%) and higher efficiency of conversion of ingesta/cocoon (18.37% to 6.71%), with respect to the control.

In light of the above observations, it was a difficult task to break the negative correlation associated with consumption and efficiency conversion on productivity traits. We concluded that polyvoltine breeds with minimum consumption index and maximum efficiency of conversion of ingesta/cocoon identified strains RMG_4_, RMW_2_, and RMW_3_ as potential polyvoltine breeding resource material for the development of nutritionally efficient breeds/hybrids in Asia and Pacific regions.

**Table 1.**
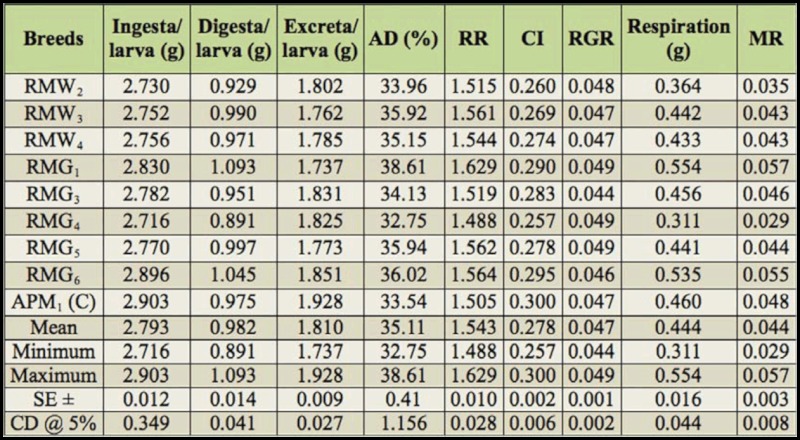
Nutrition consumption traits in polyvoltine silkworm germplasm breeds.

**Table 2.**
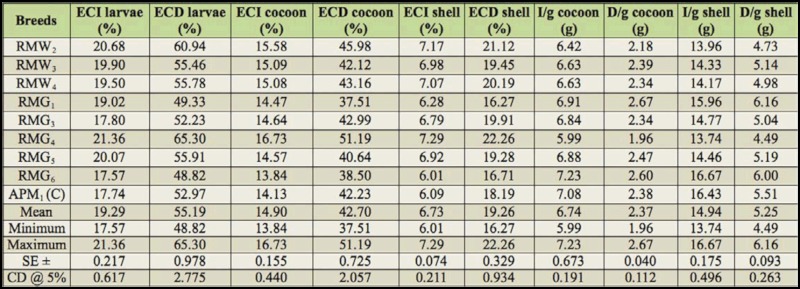
Nutritional efficiency conversion traits in polyvoltine silkworm breeds.

**Table 3.**
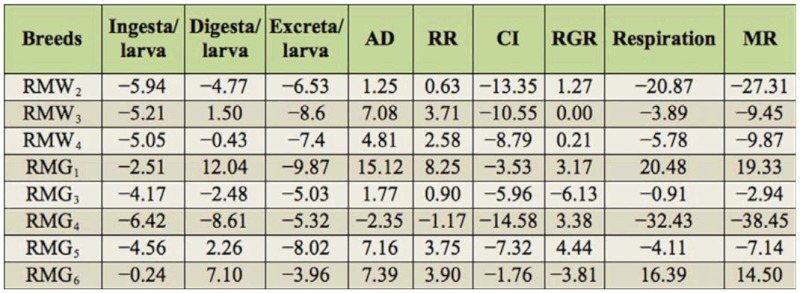
Percentage difference on nutritional traits in polyvoltine silkworm breeds over control.

**Table 4.**
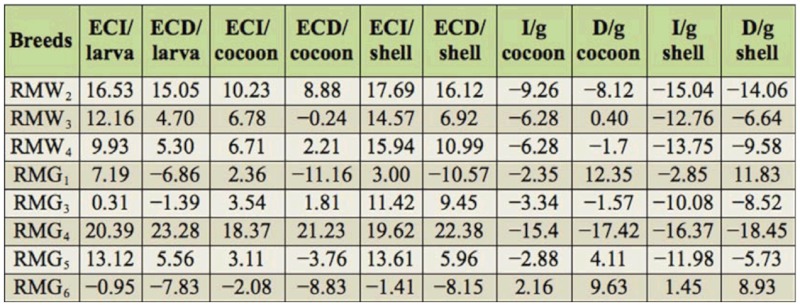
Percentage difference on nutrition efficiency conversion traits in polyvoltine silkworm breeds over control.

**Table 5.**
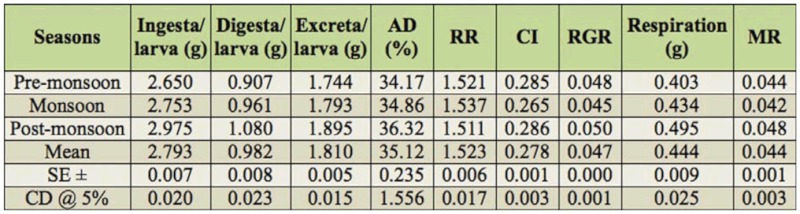
Seasonal variation on nutrition consumptional traits in polyvoltine silkworm breeds.

## **Abbreviations**

AD: approximate digestibility
Cl: consumption index
CD: critical difference
C: control
**D**/**g****cocoon**: digesta per gram cocoon
**D**/**g shell**: digesta per gram shell
ECD: efficiency of conversion of digesta
ECI: efficiency of conversion of ingesta
**l**/**g cocoon**: ingesta per gram cocoon
**l**/**g shell**: ingesta per gram shell
MR: metabolic rate
RR: reference ratio
RGR: relative growth rate
SE: standard error
